# The distribution of sensitization to common aeroallergens in patients with rhinitis and asthma in Şanlıurfa, Türkiye

**DOI:** 10.5578/tt.20239709

**Published:** 2023-09-22

**Authors:** M. Erbay

**Keywords:** aeroallergens, allergic rhinitis, asthma, skin prick test, Türkiye

## Abstract

**ABSTRACT:**

The distribution of sensitization to common aeroallergens in patients with rhinitis and asthma in Şanlıurfa, Türkiye

**Introduction:**

Sensitization to common environmental aeroallergens plays a significant role in the pathogenesis and severity of asthma and allergic rhinitis. Knowledge on the sensitization pattern helps allergen avoidance, prediction of the severity of the disease, and use of specific immunotherapy for the most common allergens. The distribution of sensitization to aeroallergens differs in every region of Türkiye. In this study, it was aimed to investigate the allergen sensitization profiles of patients with asthma and rhinitis in Şanlıurfa, which is in Southeast Türkiye.

**Materials and Methods:**

Patients with rhinitis and asthma who presented to the outpatient clinic of adult immunology and allergy between April 2021- 2022 were retrospectively evaluated. Demographic information (age, sex), rhinitis and asthma duration, location of residence, allergic and non-allergic comorbidities, smoking history and skin prick test results were extracted from medical records.

**Results:**

A total of 472 skin prick tests were performed on patients (35.4% males; 64.6% females), with a mean age of 33.8 years, and 120 (25.4%) were negative for skin reaction. The frequency of sensitivity to allergens was: grass (42.6%), cereal mixtures (41.5%), timothy grass (37.9%), cockroach (37.3%), olive tree (35%), house dust mites (Dermatophagoides farinae 27.5%, Dermatophagoides pteronyssinus 20.8%). In patients with only rhinitis (n= 305), the most frequent aeroallergen was pollen (grasses 43.6%; cereal mixtures 43.3%; timothy grass 41.6%; olive pollen 37.4%). In patients with asthma and rhinitis (n= 134), the most frequent aeroallergen was grass (44.8%). In patients with only asthma (n= 33), the most frequent aeroallergens were D. farinae (27.3%) and cockroach (27.3%).

**Conclusion:**

The most frequently detected allergens in this study were pollen, cockroach, and house dust mites, respectively. The findings revealed that pollen was the most frequent aeroallergen in subjects with allergic rhinitis with and without asthma. In patients with only asthma, the most frequent aeroallergen was house dust mites.

## Abstract (tr)

**Giriş:**

Yaygın çevresel aeroalerjenlere karşı duyarlılık, astım ve alerjik rinitin patogenezinde ve şiddetinde önemli bir rol oynar. Duyarlılık bilgisi alerjenden kaçınmaya, hastalığın ciddiyetinin tahmin edilmesine ve en yaygın alerjenler için spesifik immünoterapinin kullanılmasına yardımcı olur. Güneydoğu Anadolu’da bulunan Şanlıurfa’da astım ve rinitli hastaların alerjen duyarlılık profillerini araş- tırmayı amaçladık.

**Materyal ve Metod:**

Erişkin immünoloji ve alerji polikliniğine Nisan 2021-2022 tarihleri arasında rinit ve astım şikayetleri ile başvuran hastalar retrospektif olarak değerlendirildi. Tıbbi kayıtlardan demografik bilgiler (yaş, cinsiyet), rinit ve astım süresi, ikamet yeri, alerjik ve alerjik olmayan komorbiditeler, sigara içme öyküsü ve deri prik testi sonuçları çıkarıldı.

**Bulgular:**

Yaş ortalaması 33,8 olan hastalara (%35,4 erkek; %64,6 kadın) toplam 472 deri prik testi uygulandı ve 120 hastada (%25,4) negatif saptandı. Alerjenlere karşı duyarlılık sıklığı: otlar (%42,6), tahıl karışımları (%41,5), timothy otu (%37,9), hamam böceği (%37,3), zeytin ağacı (%35), ev tozu akarları (Dermatophagoides farinae %27,5, Dermatophagoides pteronyssinus %20.8), yakupotu (%27,5) ve sinir otu (%20,8) olarak bulundu. Sadece riniti olan hastalarda (n= 305) en sık aeroalerjen polendi (otlar %43,6; tahıl karışımları %43,3; çayırkelp otu %41,6; zeytin poleni %37,4). Astım ve rinitli hastalarda (n= 134) en sık görülen aeroalerjen çimen (%44,8) idi. Sadece astımı olan hastalarda (n= 33) en sık görülen aeroalerjenler D. farinae (%27,3) ve hamam böce- ği (%27,3) idi.

**Sonuç:**

Bu çalışmada en sık saptanan alerjenler sırasıyla polen, hamam böceği ve ev tozu akarlarıdır. Bulgular, astımı olsun olmasın alerjik riniti olan hastalarda polenin en sık görülen aeroalerjen olduğunu ortaya koydu. Sadece astımı olan hastalarda ise en sık görü- len aeroalerjen ev tozu akarlarıydı.

## INTRODUCTION

Asthma is the most common chronic respiratory illness, affecting 1-20% of the population in different countries with an estimated 300 million people worldwide ( 1 ). Aeroallergens play a key role in the pathogenesis of asthma and rhinitis. In Türkiye, the rates of allergic sensitization in adult-onset asthma are below 50% ( 1 ). Rhinitis is mainly used to describe a pattern of nasal symptoms such as nasal congestion/ obstruction, rhinorrhea, sneezing and pruritus that appear as a result of inflammation and/or dysfunction of the nasal mucosa. There are two distinct rhinitis subgroups; allergic rhinitis (AR), and non-allergic rhinitis. In our country, AR has a prevalence of 1.6- 27.5% in adults ( 2 ).

Knowledge of sensitization patterns helps allergen avoidance, prediction of the severity of the disease, and use of specific immunotherapy for the most common aeroallergens. Climate change, industrialization, global warming, the degree of environmental pollution, cultural traits, and lifestyle influence the number and types of aeroallergens to which individuals are sensitive. Many studies have shown that the spectrum of aeroallergens is significantly diverse not only between different countries but even in different parts of a country ( 3 ); therefore, it is impractical to use similar allergen avoidance measures throughout the country.

Middle Eastern countries have dry and hot summers and mild winters, which result in special vegetation with allergenic pollen that is different from allergenic pollens in Europe. Şanlıurfa is located in the southeast of Türkiye and has an approximate population of 2.1 million people (census 2021). In a study from Diyarbakır located near Şanlıurfa, grass (70.3%), wheat (46.5%) and tree (46.1%) pollens were detected as the three major allergens ( 4 ). There are limited studies in Şanlıurfa that have observed aeroallergen sensitization among patients with rhinitis and/or asthma, and the most recent study was conducted 15 years ago ( 5 ). However, changes in economic conditions, level of industrialization, and lifestyle in previous decades can influence the prevailing circulating aeroallergens and consequently affect sensitization patterns.

Therefore, this study aimed to investigate the allergen sensitization profiles of patients with asthma and/or rhinitis in Şanlıurfa. Additionally, demographic and clinical data were compared between allergic and non-allergic patients.

## MATERIALS and METHODS

### Study Design and Population

This retrospective study was conducted in University of Health Sciences Hospital, on patients aged 18 years and older who presented to the outpatient clinic of Allergy and Immunology Unit. The study was approved by the Harran University Clinical Research Ethics Committee; with registration number HRÜ/22.15.09.

The medical records of 528 adult patients who were diagnosed with rhinitis and/or asthma from April 2021 to April 2022 were retrospectively analyzed. We excluded 56 patients who applied from cities other than Şanlıurfa. Patients were diagnosed with asthma according to GINA and allergic rhinitis according to the ARIA ( 6 , 7 ). Rhinitis was defined as the presence of two or more of the following symptoms: itching, sneezing, rhinorrhea, and/or nasal obstruction for more than one hour per day ( 7 ).

Şanlıurfa, with people from different cultures, poor socioeconomic conditions, and widespread marriage between relatives is different in many ways from other regions of Türkiye. Şanlıurfa has extremely hot, dry summers and mild winters. The highest summer temperature is 46.8° C, with many sandstorms and dust storms. The economy of Şanlıurfa is mainly based on agriculture, and the main agricultural products are cotton, wheat, barley, lentils, corn, pistachios, and peppers.

Atopy is defined as IgE-mediated sensitization to at least one environmental allergen and can be detected by skin prick test. Aeroallergens are any of the various airborne substances, such as house dust mites, pollens (trees, grasses, and weeds) or fungal spores that can cause a type I- IgE-mediated allergic response. In the case of mono-sensitization, the patient is sensitized to only one allergen (allergen source), for example, timothy grass, house dust mites, or to a closely related taxonomical family or group of allergens. Poly-sensitization is sensitization to two or more allergen sources (e.g., mites, pollens, molds).

Demographic information (age, sex), rhinitis and asthma duration, location of residence, allergic (conjunctivitis, urticaria, atopic dermatitis/eczema, food allergy, drug allergy, allergy to bee venom) and non-allergic comorbidities, smoking history and skin prick test (SPT) results were extracted from medical records.

### Skin Prick Test

A standardized panel of skin prick test was performed at the clinic for all patients suspected of sensitization to aeroallergens and included 22 common aeroallergens. ALK-Abello Prick test (Hoersholm, Denmark) solution was used. The test panel used consisted of the following allergen extracts:

a. Indoor allergens: Dermatophagoides farinae (D. farinae), Dermatophagoides pteronyssinus (D. pteronyssinus), Blomia tropicalis, German cockroach, cat epithelia, dog dander, sheep dander, latex.

b. Outdour allergens: Grasses, timothy grass, cereal mixture, common ragweed, olive tree, oak, birch, Engl. plantain, mugwort, nettle.

c. Fungal allergens: Penicillium notatum, Aspergillus fumigatus, Alternaria tenuis, Cladosporium cladosporioides.

Histamine hydrochloride (10 mg/mL) and 0.9% saline were applied as positive and negative controls, respectively. The wheel diameter was measured after 20 min and reported in mm. A skin reaction of at least 3 mm greater than that produced by the negative control in the SPT was considered as a positive reaction.

### Statistical Analysis

Continuous variables were expressed as median (minimum-maximum) and mean ± standard deviation, and categorical variables were expressed as n (%). Chi-square and Fisher’s exact tests were used to compare categorical variables between the groups. Our secondary aim in the study was to observe the distribution of prevalence between those with and without a positive skin prick test in patients with a diagnosis of asthma. In their study evaluating allergic sensitization, Tantilipikorn et al. discovered that the rate of allergic sensitization was 59.4% in patients with positive SPT and 40.6% in patients with negative SPT (p< 0.001) ( 8 ). It was predicted in the study that the prevalence of asthma was the same in patients with positive and negative SPT, and if the analysis was done with G power, which is 95% confidence (1-α), 80% test power (1-β), and groups three to two patients were included, it was determined that for the actual power to be 0.801, a minimum of 134 and 93 patients in the groups, respectively, and a total of 227 patients, would be sufficient. Statistical analysis was performed using SPSS (IBM Corp. Released 2012. IBM SPSS Statistics for Windows, Version 21.0, Armonk, NY: IBM Corp.), and p< 0.05 was considered statistically significant.

## RESULTS

### Demographic and Clinical Characteristics of the Study Participants

A total of 472 skin prick tests were performed on patients, and 120 (25.4%) patients were negative for skin reaction. Of the patients, 64.6% were females (n= 305) and 35.4% were males (n= 167). Mean age was 33.8 years (standard deviation 11.4). Of the patients, 80.3% (379) lived in urban areas, and 19.7% (93) lived in rural areas. Active smoking was reported by 16.3% of the patients, 55.7% were nonsmokers, and 5.5% had stopped smoking. Allergic comorbidities were drug hypersensitivity (9.3%), conjunctivitis (8.7%), urticaria (5.7%), dermatitis (3.6%), nasal polyposis (2.8%), and celiac disease (0.8%). Hypertension (6.1%), thyroid disease (5.1%), diabetes mellitus (3%), and cancer (0.8%) were the most common comorbid diseases. Of the patients, 35.4% had asthma and 93% had rhinitis. Allergic asthma was reported in 25.6% of the patients and AR in 70.1%. Asthma and rhinitis durations were a median of six (min 0-max 40) years. In total, 305 patients (64.6%) had rhinitis without asthma, 134 patients (28.4%) had rhinitis with asthma, and 33 patients (7%) had only asthma.

### Comparison of the Data Between Allergic and Non-allergic Patients

The demographic and clinical characteristics of allergic and non-allergic patients are described in Table 1 . Atopy was significantly higher in men than in women (p= 0.04). However, there was no significant difference in comorbidities between the patient group with and without atopy.

### Skin Prick Test Results

**Figure 1:**
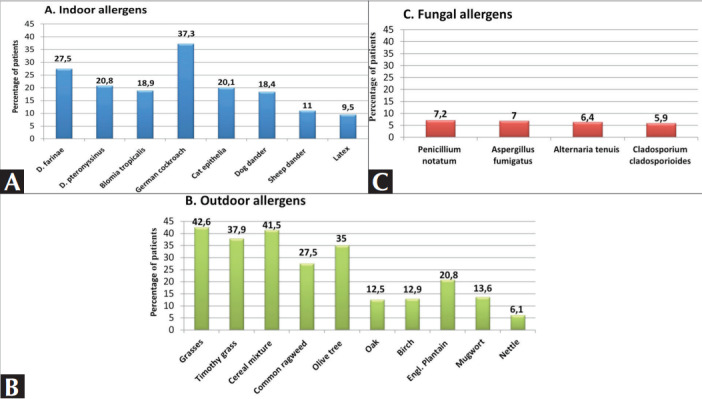
Distribution of sensitization to common aeroallergens in Şanlıurfa

The prevalence of main allergens by category is presented in Figure 1 . The most common allergen was grass [42.6% (n= 201)], followed by cereal mixtures [41.5% (n= 196)]. According to positive skin prick test results, 21.6% (76) of the patients were classified as monosensitized, and 78.4% (276) were polysensitized. Among the patients who were monosensitized, the top three allergens in frequency were grasses (35.5%), cereal mixtures (27.6%), and timothy grass (19.7%). Polysensitized patients lived in urban areas (80.5%) more than monosensitized patients (p= 0.05).

**Figure 2:**
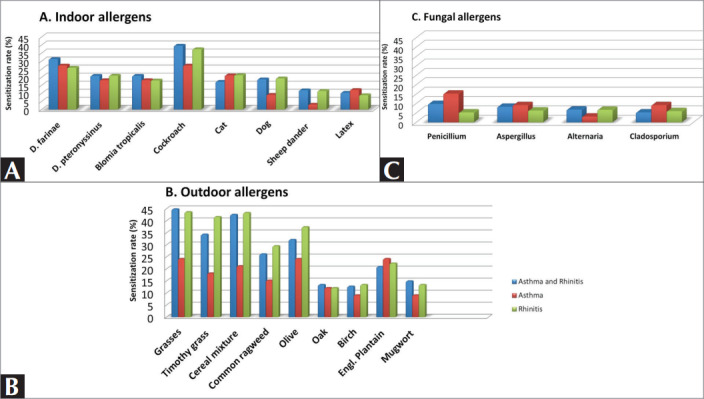
Distribution of aeroallergen sensitization according to diseases

In patients with only rhinitis, the most frequent aeroallergen was outdoor allergens (grass 43.6%; cereal mixtures 43.3%; timothy grass 41.6%; olive tree 37.4%), followed by cockroach (37.4%), common ragweed (29.5%), D. farinae (25.9%), Engl. plantain (22.3%), cat (21.3%) and D. Pteronyssinus (21%). Penicillium (5.2%) and Cladosporium (5.9%) were rare ( Figure 2 ).

In asthma patients with rhinitis, the most frequent aeroallergen was grass (44.8%), followed by cereal mixtures (42.5%), cockroach (39.6%), timothy grass (34.3%), D. Farinae (31.3%), olive tree (32.1%), common ragweed (26.1%), D. pteronyssinus (20.9%), Engl. plantain (20.9%). Nettle (5.2%) and Cladosporium (5.2%) were rare ( Figure 2 ).

In patients with only asthma, the most frequent aeroallergens were D. farinae (27.3%) and cockroach (27.3%), followed by grasses (24.2%), olive tree (24.2%), cat (21.2%), cereal mixtures (21.2%), D. pteronyssinus (18.2%), timothy grasses (18.2%), penicillium (15.2%), common ragweed (15.2%). Alternaria (3%) and sheep dander (3%) were rare ( Figure 2 ).

There was no significant difference between the patients who lived in urban or rural area regarding atopy (p= 0.25). Sensitivity to Dermatophagoides farinae and grass in urban areas (29.8% and 45.4%, respectively) was significantly higher than in rural areas (18.3% and 31.2%, respectively) (D. Farinae: p= 0.03, grasses: p= 0.01). Cockroach sensitivity was not significantly different between urban areas (36.7%) and rural areas (39.8%).

**Table 1:** Demographic and clinical characteristics in allergic and non-allergic patients with asthma and/or rhinitis

Characteristics	Total patients	Patients with positive SPT (n= 352)	Patients with negative SPT (n= 120)	p
Age (years), n (%)								
18-35	296 (62.7)	232 (65.9)	64 (53.3)	0.07
36-50	129 (27.3)	87 (24.7)	42 (35)	
51-65	40 (8.5)	27 (7.7)	13 (10.8)	
≥66	7 (1.5)	6 (1.7)	1 (0.8)	
Sex, n (%)								
Female	305 (64.6)	218 (61.9)	87 (72.5)	0.04
Male	167 (35.4)	134 (38.1)	33 (27.5)	
Smoking, n (%)	77 (16.3)	53 (19.7)	24 (24.7)	0.3
Residence location, n (%)				
Urban	379 (80.3)	287 (81.5)	92 (76.7)	0.25
Rural	93 (19.7)	65 (18.5)	28 (23.3)	
Allergic comorbidities, n (%)				
Conjunctivitis	41 (8.7)	33 (9.4)	8 (6.7)	0.36
Urticaria	27 (5.7)	22 (6.3)	5 (4.2)	0.4
Dermatitis	17 (3.6)	15 (4.3)	2 (1.7)	0.15*
Drug allergy	44 (9.3)	34 (9.7)	10 (8.3)	0.7
Non-allergic comorbidities, n (%)				
Hypertension	29 (6.1)	20 (5.7)	9 (7.5)	0.47
Diabetes	14 (3)	11 (3.1)	3 (2.5)	0.51*
Thyroid disease	24 (5.1)	15 (4.3)	9 (7.5)	0.16
Asthma, n (%)	167 (35.4)	121 (34.4)	46 (38.3)	0.43
Rhinitis, n (%)	439 (93)	331 (94)	108 (90)	0.13

SPT: Skin prick test. *Fisher’s exact test

## DISCUSSION

This study is very significant due to being the largest patient population study evaluating the sensitization rate of aeroallergens in Şanlıurfa. The most frequently detected allergens in this study were pollen, followed by cockroach, and house dust mite. Another important finding of the study is the most frequent aeroallergens were house dust mite in patients with only asthma.

In our study, atopy was significantly higher in men than in women. Similarly, several studies suggest that, as both children and adults, atopy is more common in males than females ( 9 ). Furthermore, women exhibited a lower prevalence of aeroallergen sensitization than men in a study conducted as a part of Polish Epidemiology of Allergic Diseases study ( 10 ). However, in Canadian and US studies, no significant differences have been observed in the frequency of atopy between males and females ( 11 , 12 ).

In our study, no significant difference in atopy prevalence was found between rural and urban regions. Rural populations are reported to have less atopic sensitization and disease than urban dwellers as they may be protected by exposure to high levels of microbes during infancy and childhood ( 13 ). Chan-Yeung et al. have demonstrated that a rural city in Canada had the lowest risk of atopy and sensitization to almost all specific allergens ( 14 ). Urban children are often exposed and sensitized to multiple indoor allergens, including cockroaches, dust mites, and mold. In our study, sensitivity to D. farinae and grasses in urban areas (29.8% and 45.4%, respectively) was significantly higher than in rural areas (18.3% and 31.2%, respectively). Similarly, İrani et al. have reported that living in a city increased the odds of sensitization to both pollen and dust twofold ( 15 ). It is understandable that house dust mite sensitivty is high in the urban area because it is an indoor allergen. What is causing the high pollen sensitivity in urban areas? This can be explained by increased ambient CO2 which may cause some plant products to become more allergenic, resulting in higher sensitization ( 16 ). Furthermore, another study revealed that the higher CO2 concentrationsin urban areas resulted in ragweed that produced significantly higher amounts of pollen ( 17 ). The fact that urban and rural areas are mostly interconnected in Şanlıurfa may be one of the factors leading to higher grasses sensitivity in urban areas.

Grass pollen is common in warm regions with low humidity. The hot and dry climate of the Şanlıurfa region, which is predominantly agricultural, explains the high frequency of detection of sensitization to grass pollens on skin tests in our study. In previous studies in the regions of Malatya (48.9%), Adıyaman (77.5%), and Diyarbakır (70.4%), which have a similarly hot and dry climate, sensitivity to pollen (grass and cereal mixtures) has been most frequently reported ( 4 , 18 , 19 ). In another study in Şanlıurfa (n= 38), the most common allergen sensitivities have been found as grass (39.5%) and cereal mixtures (34.2%) ( 20 ).

In studies from different regions of Türkiye, sensitivity rates for house mites D. pteronyssinus and D. Farinae were 69% in the Mediterranean region, 50% and 44% in Bursa, and 84% and 78.2% in the Eastern Black Sea region, respectively ( 21 , 22 , 2321-23 ). In contrast, in our study, we observed lower sensitivity to house mites D. Pteronyssinus (20.8%) and D. Farinae (27.5%). Globally, house dust mite are common sensitizing aeroallergens in patients with AR. In our study for patients with only AR, the most common aeroallergen was pollens (grasses, cereal mixtures, timothy grass, olive pollen) followed by cockroach, common ragweed, and D. farinae. House dust mite allergy is more common in humid coastal areas ( 24 ). The reason why house dust mite sensitivity was low in our study might be because the region has a dry climate and is not located near the coast. House mite sensitivity has been found to be low in studies in Malatya (40.2%) and Mardin (16.9%), which have similar climatic conditions to Şanlıurfa ( 18 , 25 ). Therefore, our results are consistent with some previous studies that indicated grass pollens as the most common allergen in patients with AR ( 21 , 26 ). Sensitization to indoor allergens has been described as the main allergen category in asthma, causing bronchial hyperreactivity and uncontrolled disease ( 27 ). Although we did not find high mite sensitivity due to the dry, hot climate, the most frequent aeroallergens D. farinae (27.3%) and cockroach (27.3%) were found only in asthma patients in our study. Therefore, house mite is an important allergen for asthma regardless of climate.

Cockroaches are more common in places with poor socioeconomic status and living conditions ( 28 ). While cockroach sensitivity was found to be high (37.3%) in our study, low sensitivity was found in İstanbul (8%) and the Aegean region (5.3%) ( 29 , 30 ). In general, for Southeast Asian nations, AR is associated with cockroach sensitization ( 31 ). In our study, cockroach sensitization was reported at 37.4%, 27.3%, and 39.6% in patients with rhinitis, only asthma, and asthma patients with rhinitis, respectively. Increased sensitivity to cockroaches may be related to crowded housing, poor hygiene, and low socioeconomic status, which is common in Şanlıurfa.

Olea europaea (olive tree) cultivation is concentrated around the towns of Aydın, İzmir, Muğla, Balıkesir, Bursa, Manisa, Çanakkale, Gaziantep, and Mersin and in the regions of the Aegean, Marmara, and Southeast Anatolia. There is a high level of olive cultivation and many olive trees in Şanlıurfa. In our study group, sensitivity to olive tree pollen was high at 35%. In a study in Bursa, one of the centers of olive cultivation in Türkiye, there was high olive tree sensitivity (33.2%), similar to our study ( 21 ). In a study by Terzioğlu et al. in İstanbul, sensitivity to olive tree pollen (11%) has also been found to be lower ( 32 ).

There are several limitations to this study. First, this is a real-world, retrospective study; therefore, further prospective studies should be conducted on a larger scale to determine the incidence and prevalence of allergen sensitization in Southeast Türkiye. Second, this study was primarily a single-center study, however, sample population was large. Third, aeroallergen sensitization was not assessed along with allergen concentrations in the atmosphere. The accuracy of the skin prick test sensitivity was 85% in the study by Nevis et al, while Alternaria’s sensitivity was 42% ( 33 ). The false positivity of inhalant allergen sensitivity in polysensitized individuals could not be adequately detected in our study due to the lack of component-resolved allergy diagnosis or a nasal provocation test.

## CONCLUSION

Our findings revealed that pollen was the most frequent aeroallergen sensitization in subjects with AR with and without asthma. In patients with only asthma, the most frequent aeroallergen was house dust mite. From this study, we learned several lessons. First, because the population of Şanlıurfa is sensitized to a wide variety of aeroallergens, we do not believe that it is appropriate to test for allergies to only a small number of aeroallergens. Second, because common aeroallergens are different for each region, the region should be considered when composing an aeroallergen panel. The findings of our study may be applicable in other provinces in Southeast Türkiye that have similar environmental factors to Şanlıurfa, including climate, geography, seasonal variation, and lifestyle. We hope that the results will be useful for health system policy makers when planning respiratory allergic disease prevention programs in the region. Moreover, Göbekli Tepe, located in the province Şanlıurfa in Southeastern Anatolia, is one of the most ancient farming areas in the world, where people lived in Upper Mesopotamia about 11.500 years ago and it is frequently visited by tourists. Our study provides recommendations on aeroallergens in Middle Eastern countries to allergists who are often required to give advice to patients who plan on traveling to the region.

## Ethical Committee Approval

This study was approved by the Harran University Clinical Research Ethics Committee (Decision no: 22.15.09, Date: 08.08.2022).

## CONFLICT of INTEREST

The authors declare that they have no conflict of interest.

## AUTHORSHIP CONTRIBUTIONS

Concept/Design: ME

Analysis/Interpretation: ME

Data acqusition: ME

Writing: ME

Clinical Revision: ME

Final Approval: ME
